# Salivary Glands in Predatory Mollusks: Evolutionary Considerations

**DOI:** 10.3389/fphys.2017.00580

**Published:** 2017-08-10

**Authors:** Giovanna Ponte, Maria Vittoria Modica

**Affiliations:** ^1^Department of Biology and Evolution of Marine Organisms, Stazione Zoologica Anton Dohrn Napoli, Italy; ^2^Association for Cephalopod Research - CephRes Napoli, Italy; ^3^Department of Integrative Marine Ecology, Stazione Zoologica Anton Dohrn Napoli, Italy

**Keywords:** molluscs, gastropods, cephalopods, predatory strategies, adaptations, evolution, salivary glands

## Abstract

Many marine mollusks attain or increase their predatory efficiency using complex chemical secretions, which are often produced and delivered through specialized anatomical structures of the foregut. The secretions produced in venom glands of *Conus* snails and allies have been extensively studied, revealing an amazing chemical diversity of small, highly constrained neuropeptides, whose characterization led to significant pharmacological developments. Conversely, salivary glands, the other main secretory structures of molluscan foregut, have been neglected despite their shared occurrence in the two lineages including predatory members: Gastropoda and Cephalopoda. Over the last few years, the interest for the chemistry of salivary mixtures increased based on their potential biomedical applications. Recent investigation with -omics technologies are complementing the classical biochemical descriptions, that date back to the 1950s, highlighting the high level of diversification of salivary secretions in predatory mollusks, and suggesting they can be regarded as a pharmaceutical cornucopia. As with other animal venoms, some of the salivary toxins are reported to target, for example, sodium and/or potassium ion channels or receptors and transporters for neurotransmitters such as, glutamate, serotonin, neurotensin, and noradrenaline, thus manipulating the neuromuscular system of the preys. Other bioactive components possess anticoagulant, anesthetic and hypotensive activities. Here, we overview available knowledge on the salivary glands of key predatory molluscan taxa, gastropods, and cephalopods, summarizing their anatomical, physiological and biochemical complexity in order to facilitate future comparative studies on main evolutionary trends and functional convergence in the acquisition of successful predatory strategies.

## Introduction

Predation is a complex habit involving morphological, physiological, neural, and behavioral adaptations. Such lifestyle evolved multiple times in almost all molluscan classes, including the sessile Polyplacophora and Bivalvia. The veiled chiton *Placiphorella velata* uses its head flap and precephalic tentacles to capture small invertebrates (McLean, 1962), while in the bivalve order Anomalodesmata most of the species engulf small crustaceans with their eversible inhalant siphon (e.g., Morton, 1981, 1984). Apart from these remarkable cases it is undoubtable that some lineages of Gastropoda and the whole class Cephalopoda fully exploited the opportunities offered by an active predatory lifestyle.

In Gastropods, and besides some “archaeogastropods,” a predatory lifestyle evolved independently several times in a number of Caenogastropoda lineages, including (i) Neogastropoda (with about 40 families), (ii) Tonnoidea, (iii) Naticoidea, and (iv) Ficoidea. Fossil record suggests that predation evolved almost simultaneously in late Cretaceous, in the framework of the major reorganization of marine communities known as the Mesozoic marine revolution (Vermeij, 1977; Taylor et al., 1980; Tracey et al., 1993).

In contrast, Cephalopods are all carnivorous, coleoids being macrophagous predators, and *Nautilus* a scavenger. They emerged as predators since their major diversification event in middle-upper Paleozoic (Kröger et al., 2011) and evolved sophisticated techniques to search, capture, and kill their preys.

Both in Gastropods and in Cephalopods the physiology and sensory abilities allow the animals to seek diverse preys through a variety of feeding behaviors and predatory strategies (Hanlon and Messenger, 1996; Rodhouse and Nigmatullin, 1996; Modica and Holford, 2010). Their digestive system is arranged to form several compartments with morphological, structural, and functional specializations (Mangold and Bidder, 1989; Fretter and Graham, 1994). Among the anatomical and physiological adaptations of the digestive system enabling predation in these animals, a primary role can be attributed to the salivary glands. These discharge their secretion, via connecting ducts, into the buccal cavity, that—due to the morpho-functional characteristic of the molluscan *Bauplan*—corresponds to the immediate proximal space inside the mouth, allowing the closest proximity to the prey.

While earlier studies attributed only a lubricant role to salivary secretions, it is since the late nineteenth century that evidences begun to accumulate on the ability of salivary glands to produce bioactive substances. Modern approaches, including—omics technologies, have been confirming the high diversification of salivary secretions in predatory molluscs, identifying them as a very promising and still neglected taxon for the discovery and characterization of novel bioactive compounds.

Here, we summarize the available knowledge on salivary glands and their specialization in gastropods and cephalopods, in order to offer a framework to further detailed comparative studies aiming to elucidate how successful predatory strategies emerged in different molluscan lineages.

## Morphology and organization of the salivary glands

Salivary glands are generally acinous in Caenogastropoda, but their anatomy and organization varies greatly, even in predatory taxa (Fretter and Graham, 1994). Evidences from ontogenetic comparative studies suggest that they may be considered homologous at least in “higher” Caenogastropods (Page, 2000).

**Figure 1:**
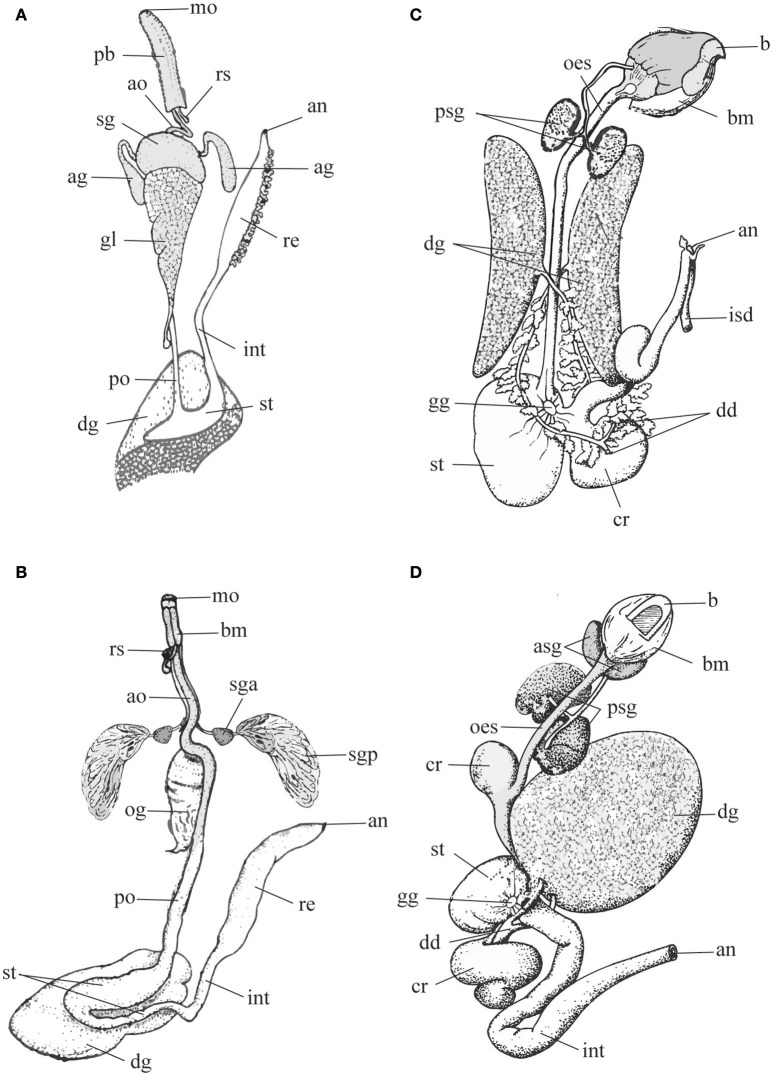
Schematic representation of digestive tract in predatory gastropods **(A,B)** and cephalopods **(C,D)** to highlight differences in the morphology and arrangements of the salivary glands (gastropods: sga, sgp or sg; cephalopods: asg, psg). Left panel, Gastropods. **(A)** Neogastropoda Muricidae (modified after Wu, 1965); **(B)** Tonnoidea (modified after Barkalova et al., 2016). Right panel, Cephalopods. **(C)** *Sepia*, and **(D)** *Octopus* (modified after Budelmann et al., 1997). Terms and abbreviations follow original descriptions and despite analogies are not synonymized here. ag, accessory salivary gland; an, anus; ao, anterior esophagus; asg, anterior salivary gland; b, beak; bm, buccal mass; cr, crop; dd, digestive duct; dg, digestive gland; gg, gastric ganglion; gl, gland of Leiblein; int, intestine; isd, ink sac duct; mo, mouth; oes, esophagus; og, oesophageal gland; pb, proboscis; po, posterior esophagus; psg, posterior salivary gland; re, rectum; rs, radular sac; sg, salivary gland; sga, anterior lobe of the salivary gland; sgp, posterior lobe of the salivary gland; st, stomach.

Tonnoidea possess a single pair of large salivary glands differentiated in anterior and posterior lobes (see Figure 1B) that in the different families can be either morphologically separated or undivided, but with a proximal region resembling the anterior lobe. The anterior and posterior lobes discharge their secretion via a common duct (Barkalova et al., 2016), a characteristic that supports the interpretation of the two lobes as parts of a single gland. The anterior lobe is generally small and can be tubular or acinous, while the posterior lobe is more conspicuous, consists in morphologically uniform, radially arranged blind tubules and secretes sulfuric acid (Weber, 1927; Houbrick and Fretter, 1969; Fänge and Lidman, 1976; Hughes and Hughes, 1981; Andrews et al., 1999).

In Neogastropoda, which are almost all predators, both primary and accessory salivary glands are present. Primary salivary glands are acinous, generally paired and constituted of narrow branched ducts with a small lumen. They are joined in a single glandular mass in some species, but separate ducts are always maintained. The thin salivary ducts run along the esophagus until opening into the roof of the buccal cavity (Figure 1A). The secretory epithelium comprises two mixed cell types: superficial ciliated cells secreting mucus, and basal cells with apocrine secretion. The secretion is delivered through ciliary movement, as the outer muscular layer is poorly developed (Andrews, 1991). The accessory salivary glands are present in the basal family Cancellariidae, supporting the hypothesis that they are a synapomorphy of the Neogastropoda. Anyway, they are reduced to a single gland or absent in a number of families (Ponder, 1973; Andrews, 1991); even in families where they are generally well-developed (e.g., in Muricidae) cases of secondary loss are frequent. In most neogastropods accessory salivary glands are tubular and consist of a columnar secretory epithelium surrounded by a richly innervated sub-epithelial muscular coat. Gland cells, producing a peculiar granular secretion, lie outside the muscle layer and open via long necks in the central lumen of the gland, from which the secretion is delivered at the tip of the buccal cavity with non-ciliated ducts (Ponder, 1973; Andrews, 1991).

In cephalopods, three types of salivary glands are associated with the buccal mass: the submandibular gland, and the paired anterior and the posterior salivary glands (Mangold and Bidder, 1989; Budelmann et al., 1997).

The submandibular (or sublingual) gland, a non-paired organ lying below the salivary papilla and arranged into several lobes, is well-developed in octopods and *Vampyroteuthis* but reduced to small folds in *Nautilus*.

The paired anterior salivary glands are larger than the former, made by ramified tubules, and variable in different species. In *Nautilus* and cuttlefishes they are enclosed in the musculature (lateral lobes), while in octopuses they lay at both sides of the buccal mass (Figures 1C,D).

The posterior salivary gland lies behind the buccal mass. It is missing in nautiloids, paired in cuttlefishes and octopuses and unpaired in teuthoids and *Cirroteuthis*. It consists of numerous lobules made-up by tubules producing viscous secretions that are transported by muscular ducts to a common terminal canal opening into the anterior buccal cavity, nearby the apex of the salivary papilla (Mangold and Bidder, 1989).

In the posterior salivary gland two types of epithelia have been described. In type A, polarized columnar cells containing few mitochondria are responsible of apocrine secretions. Type B, restricted to the duct area, is characterized by three types of cells, the most important being striated with abundant mitochondria and microvilli involved in active ion transport and excretion (Budelmann et al., 1997).

This assembly, typical of *Octopus* and *Eledone*, is simplified in *Sepia* where a single type of secretory cells, corresponding to *Octopus* type A is found.

The three salivary glands play different roles in feeding. The submandibular gland contributes to lubricating the passage of the food, the posterior salivary glands produce secretions used to paralyze the prey within a few seconds after capture (Ghiretti, 1959, 1960), while the secretion of the anterior salivary gland facilitates the action of the very viscous secretions of the posterior salivary glands.

## Physiology of the salivary glands

Little is known about the nervous control of salivary secretion in molluscs: the most accurate review is provided by House (1980), and more recent updates are missing. Here, we summarize available knowledge on the topic to facilitate the understanding of its evolutionary relevance.

According to House (1980), the following sequence of events occurs in the salivary glands of several invertebrate taxa, not limited to molluscs: (i) neurohormone or transmitter release, (ii) receptor activation in gland cell, (iii) build-up of second messenger, (iv) electrical events (i.e., ion channels open, membrane potential change) activated by the receptor activation in the gland cell often synergistically to the build-up of second messenger; (v) secretory events (i.e., enzyme release, ion, and fluid secretion) initiated by the build-up of the second messenger. Evidence for a direct initiation of secretory events from the electrical ones appear possible, but research is missing (House, 1980; Barber, 1983). Besides the changes in membrane potential and conductance, neuro-modulators or neurotransmitters may provide uncoupling of neighboring gland cells, thus providing further regulation of the secretory event (House, 1980).

In gastropods, the neural control of salivary glands is quite simple (House, 1980). A resting potential is shown by secretory cells and not by muscle fibers in the gland. In fact, an electrogenic sodium pump distributes potassium ions, giving to the cell basal membrane physiological properties similar to those of certain muscle and nerve cells. High level of coupling is observed, and therefore synchronous, spontaneous action potentials are generated, resulting in an all-or-none action potential response. Studies on the ionic basis of the action potential indicate that the inward current is carried chiefly by calcium ions with a minor contribution due to sodium. Calcium entry triggers the exocytosis of granules from the cells. Because of coupling, the number of intervening cells alters the delay between the recorded action potentials, and spontaneous miniature depolarizations promote further massive release from the gland.

In contrast, the posterior salivary glands of cephalopods are known for their abundant innervation (at least 30,000 axons from the salivary nerves reach the glands, and about 10,000 axons in the salivary duct nerves control the muscular contraction of the duct; Young, 1965; review in House, 1980).

A dual innervation is reported for the posterior salivary gland (House, 1980). Larger axons originating from the subradular ganglia innervate circular smooth muscle cells surrounding the tubules. The neuromuscular junctions show membrane thickenings and at the nerve endings many small agranular vesicles and some large ones (predominantly cholinergic) are present. The muscle cells in the salivary duct receive innervation from presynaptic fibers that contain a heterogeneous population of vesicles (mostly monoamines).

The second innervating component consists of smaller axons derived from cell bodies in the superior buccal lobe (supra-oesophageal mass, part of the “brain”). These axons end close to the basal membranes of the tubular cells, with a synaptic cleft (20 nm) and apparently no synaptic membrane specializations. These nerve endings contain a mixed population of vesicles (i.e., small agranular vesicles, dense-cored, and granular vesicles), where catecholamines are found. Noradrenaline and 5-HT are considered to be transported along axons toward the glands from cell bodies in the superior buccal lobe. In analogy, and due to significant quantities of octopamine and tyramine found in the superior buccal lobe (Juorio and Molinoff, 1971; Juorio and Philips, 1975; Ponte and Fiorito, 2015), these amines appears to be transported to the glands where they are released on nerve stimulation.

## Biochemical complexity of salivary secretions

Research on molluscan bioactive compounds have been mostly focused on cone snails, which are among the most studied and best understood of all venomous animals, and led to the pharmacological development of one commercially available drug (the ziconotide, a Ca^2+^ channel blocker) plus other compounds that are now in pre-clinical trials. Despite the discovery of alpha-conotoxins in the salivary secretion of *Conus pulicarius* (Biggs et al., 2008), in Conoidea toxin production is mostly due to venom gland, a synapomorphy of this superfamily evolved from the mid-esophageal gland of Leiblein (Ponder, 1973).

Studies on the biochemical properties of salivary secretion in other predatory molluscs are extremely reduced and mostly outdated (see Table 1 for a summary).

**Table 1:** A tabularized overview of cytolytic, hypotensive, neurotoxic and other enzymatic substances discovered as product of salivary glands of some gastropod and cephalopod species (not exhaustive list).

		**Cytolitic**		**Hypotensive**			**Neurotoxins**					**Other enzymes**						
		**Echotoxins**	**n/a**	**Eleidosin**	**Oct-TK**	**Unknown**	**CAP**	**Cephalotoxin**	**Conotoxins**	**ShK toxins**	**Unknown**	**Carboxypeptidase**	**Chitinase**	**Hyaluronidase**	**Metalloprotease**	**Pacifastin**	**Phospholipase A2**	**S1 peptidase**
**Gastropoda**	*Acanthinucella spirata*					✓					✓							
	*Nucella lapillus*					✓					✓							
	*Stramonita haemastoma*					✓					✓							
	*Neptunea antiqua*										✓							
	*Cumia reticulata*	✓					✓		✓	✓		✓			✓			✓
	*Nassarius* spp.										✓							
	*Conus pulicarius*								✓									
	*Monoplex parthenopeum*	✓																
**Cephalopoda**	*Sepia latimanus*		✓				✓				✓		✓				✓	✓
	*Sepia pharaonis*		✓				✓	✓					✓		✓	✓	✓	✓
	*Sepia esculenta*		✓					✓										
	*Sepioteuthis australis*		✓				✓	✓				✓	✓		✓	✓	✓	✓
	*Uroteuthis noctiluca*		✓				✓					✓	✓		✓	✓	✓	✓
	*Octopus cyanea*		✓				✓					✓	✓	✓		✓		✓
	*Octopus vulgaris*		✓		✓			✓										
	*Octopus kaurna*		✓		✓		✓				✓		✓			✓		✓
	*Abdopus aculeatus*		✓										✓		✓	✓		✓
	*Hapalochlaena maculosa*		✓								✓	✓	✓	✓		✓		✓
	*Eledone moschata*		✓	✓														
	*Eledone cirrhosa*		✓	✓														
	*Pareledone turqueti*		✓										✓					✓
	*Adelieledone polymorpha*		✓										✓					✓

*Data from the following literature and references within: Biggs et al., 2008; Fry et al., 2009a,b; Modica and Holford, 2010; Ruder et al., 2013; Modica et al., 2015. See text for details*.

In Gastropoda, a complex salivary secretion containing different toxins is reported for several Tonnoidea (Andrews et al., 1999; Barkalova et al., 2016), including neurotoxins and cytolytic-hemolytic echotoxins (Shiomi et al., 1994). Additionally, sulfuric acid bringing the saliva to a pH of 2 or less has been detected in nearly all tonnoideans (Barkalova et al., 2016).

In Neogastropoda a high quantity of tetramine, histamine, choline, and choline esters has been reported in whelks' salivary glands (Endean, 1972; Shiomi et al., 1994; Power et al., 2002). Tetramine blocks nicotinic acetylcholine receptors (Emmelin and Fänge, 1958) and has been responsible of a number of human intoxications (e.g., Fleming, 1971; Reid et al., 1988). In addition, salivary secretions of whelks include still unidentified inhibitors of neuronal Ca^2+^ channels (e.g., in *Neptunea antiqua*, Power et al., 2002). Cystein-rich glycoproteins were detected in some Nassariidae and Muricidae (Martoja, 1971; McGraw and Gunter, 1972; Minniti, 1986; Fretter and Graham, 1994). These may account for the observed effects of salivary secretion, including: (i) flaccid paralysis in *Mytilus edulis* and in barnacles (Huang and Mir, 1972; Carriker, 1981; Andrews, 1991; Andrews et al., 1991; West et al., 1998); (ii) decrease of cardiac activity, vasodilatation, hypotension, and smooth muscle contraction in mammals (Huang and Mir, 1972; Hemingway, 1978) (iii) disruption of neuromuscular transmission in rat (West et al., 1998). In some Volutidae, the accessory salivary glands produce a narcotizing compound, with a very low pH, inducing muscular relaxation in the preys (Bigatti et al., 2009).

Besides these earlier studies, the only modern transcriptomic approach applied for the characterization of salivary secretion in a non-conoidean gastropod has been carried out on the hematophagous *Cumia (Colubraria) reticulata*, revealing a remarkable complexity of the salivary secretion. Neurotoxins, echotoxins, and several enzymes were detected, as well as putative inhibitors of hemostasis such as, TFPI-like protease inhibitors, the novel VWFA1 domain-containing proteins and ENPP-5 (Modica et al., 2015).

In Cephalopoda the posterior salivary gland is responsible for the production of a number of different biologically active substances, while the anterior salivary glands release large amount of mucus containing neutral glycoproteins (SH, S-S groups) and sialic acid, dipeptidase, and hyaluronidase, that probably facilitate the delivery of the viscous secretions of the posterior salivary gland and may be involved in external pre-digestion (e.g., Furia et al., 1975; Nixon, 1984; Hernández-García et al., 2000).

The toxic effects of posterior salivary glands secretion in Octopoda (including irreversible paralysis and death in crustaceans) were recognized in the late nineteenth century by Lo Bianco (1888). Toxicity was firstly attributed to the numerous biogenic amines produced by the posterior gland, including tyramine, histamine, acetylcholine, octopamine, and serotonin. Subsequently this was accounted to a protein component (Songdahl and Shapiro, 1974) named cephalotoxin (Ghiretti, 1959, 1960). In *Octopus vulgaris* two heavily glycosylated cephalotoxins, alpha and beta, have been characterized (Cariello and Zanetti, 1977), while a divergent SE-cephalotoxin was isolated from *Sepia esculenta* by Ueda et al. (2008). Reported effects of cephalotoxins include inhibition of respiration in crabs, inhibition of blood coagulation in both crabs and humans, and paralysis of crabs and cockroaches (Ghiretti, 1960).

Several hypotensive compounds have been also identified, including tachykinins such as, Eledoisin (Anastasi and Erspamer, 1962), originally isolated from *Eledone aldrovandi* and *Eledone moschata*, OctTK-1 and OctTK-2 from *O. vulgaris* (Kanda et al., 2003) and an OctTK-1 homolog from *Octopus kaurna* (Fry et al., 2009a).

CAP proteins have been detected in several cephalopod species, as well as novel putative toxins with no homology to any known peptide type (Fry et al., 2009a).

The active components of the posterior salivary gland secretion include also a range of enzymes identified in a number of cephalopods species, including S1 peptidase, hyaluronidase, carboxypeptidase, metalloprotease, phospholipase A2 (Romanini, 1952; Grisley and Boyle, 1990; Grisley, 1993; Fry et al., 2009a; Ruder et al., 2013).

Despite their potential, most of toxicological research in cephalopods has been focused on the TTX-like compounds produced by *Hapalochlaena*, which are responsible of human fatalities. *Hapalochlaena* TTX is not an endogenous salivary toxin, as it is produced by endosymbiotic bacteria in the salivary glands and in other parts of the body of the animal (Yotsu-Yamashita et al., 2007).

## Concluding remarks

Many competing hypothesis have been proposed for the phylogenetic relationships of the Mollusca, using morphological, molecular, and other characters (see Sigwart and Lindberg, 2015 for a critical review). According to the most recent reconstruction of evolutionary relationships of Molluscs (Smith et al., 2011), gastropods and cephalopods are paraphyletic, implying that a predatory lifestyle was independently acquired in these two well-diversified lineages.

Morphology of salivary glands displays different patterns in Gastropoda and in Cephalopoda. Cephalopods share a common arrangement, with a great uniformity in all the Coleoidea so far studied and minor variations (as expected) in *Nautilus*, congruently with the hypothesis that a carnivorous or predatory lifestyle is an ancestral characteristic of the group. Conversely, predatory gastropods developed a number of different morphological arrangements despite some shared characteristics, as expected in a group that evolved predation more recently multiple times in at least three main lineages, from an ancestral microphagous feeding ecology.

The remarkably higher complexity of the physiological regulation of salivary secretion in Cephalopoda compared to Gastropoda further confirms a major commitment toward predation since the early evolutionary history of the former group. In gastropods, the basic physiology of salivary secretion appears in agreement with a plesiomorphic condition of microphagous feeding.

If we consider the bioactive compounds secreted in the salivary glands of both groups, it should be noted that cephalopods evolved characteristic enzymes and neuropeptides belonging to families that are shared with many other venomous taxa, including snakes and spiders (Fry et al., 2009a,b; Casewell et al., 2013), while the reduced number of gastropods studied so far display a great inter-lineage variability and a reduced number of shared compounds with non-molluscan lineages. This condition, if confirmed by further studies on a broader range of taxa likely reflects independent evolution of predation in the different lineages of predatory Caenogastropods.

In summary, while in gastropods the onset of a predatory adaptation evolved recently (in the late Cretaceous) with respect to their evolutionary origin that dates back to the late Cambrian and had to cope with a *Bauplan* built for microphagy, cephalopods evolved predation from a scavenger ancestor at the time of their major diversification in middle-upper Paleozoic (Kröger et al., 2011).

In spite of the differences of salivary glands of gastropods and cephalopods we simplified in this review, a common feature emerged: the presence of multiple glands corresponding to an extremely rich chemical assemblage. This trait may have facilitated the specialization and differentiation of different cellular districts to achieve the final composition of the saliva. Several bioactive salivary components with cytolytic, hypotensive and, above all, neuroactive activity are excellent candidates for biotechnological development, due to millions years of natural selection that have contributed to their specificity, a key factor in the evolutionary success of these predatory mollusks.

## Author contributions

GP and MM developed the concept of the manuscript, searched the literature and wrote a draft. MM further developed the early draft. GP elaborated the figure.

### Conflict of interest statement

The authors declare that the research was conducted in the absence of any commercial or financial relationships that could be construed as a potential conflict of interest. The reviewer CM-S and handling Editor declared their shared affiliation, and the handling Editor states that the process met the standards of a fair and objective review.
